# Medical Opinion and Movement

**Published:** 1906-12-01

**Authors:** 


### Medical Opinion and Movement.

Medical men certainly have a right to protest against motor-cars employed for professional purposes being taxed on the same scale as pleasure cars. We understand that in France this injustice is not perpetrated. On registering the car the physician has but to state that he is using it for professional purposes and he obtains a 50 per cent. reduction on the usual tax levied. By combined action and representation to the proper quarter the profession in England should soon secure that the tax on their motors shall be regulated on some such system as obtains in France.

It is satisfactory to note that the County Council is applying at the next Parliamentary session for further powers to control the milk-supply to the Metropolis. There is no more serious question touching the health of a community than the provision of a pure milk. Under existing conditions milk which has been condemned locally finds an easy access to the London market. In order to check this wholesale supply of impure milk from the country, the Council is asking for special powers for taking samples of milk at railway stations, and also for requiring lists from cow-keepers, dairymen, and vendors of milk of their sources of milk-supply.

A well-attended meeting of medical men was recently held at the Fulham Town Hall to consider the question of hospital abuse. The following resolution was put to the meeting and carried unanimously:—

> That in the opinion of this meeting grave hospital abuse exists, in that patients who can afford to pay for treatment obtain advice and medicines free, thereby funds being wasted that have been subscribed for the deserving poor; that this abuse is growing, and that it is desirable, both in the interests of the poor, and of the subscribers, and of the medical profession, that it should cease.

Many excellent speeches were made in support of the resolution, and much evidence was adduced to show the prevalence and extent of the abuse. Dr. Ford Anderson struck the keynote of the situation by maintaining, as we have so frequently urged in these columns, that "the profession must be convinced, united, and determined," in order to attain their objects and remedy the evil.

Within the next fortnight medical practitioners throughout the United Kingdom will be asked to record their votes for the election of their Direct Representatives on the General Medical Council. There are ten candidates offering themselves for election to the three seats for England and Wales, and if the electors will carefully peruse the addresses of the candidates in the medical papers, they should have no difficulty in choosing those who will be most likely to further the interests of the profession on the Council. There are many questions calling for the urgent attention of the General Medical Council, including the question of increased representation of general practitioners on the Council. It is to be hoped that medical men all over the country will evince their conviction of the importance of these matters and show their appreciation of their representation on the Council by duly recording their votes. The apathy of the profession shown at previous elections has been used as an argument against any proposal for increased representation. Let at least this line of argument be removed by the coming election. A heavy poll will do more than anything else can to strengthen the hands of the elected representatives, and to enable them to carry out the measures of reform to which they are pledged. It will be a great source of regret to many that through some inadvertence Mr. Rutherford Morison's nomination paper was sent in too late, and he has therefore failed to be nominated as a candidate.

In reply to a deputation from the National Conference on Infant Mortality the Premier and the President of the Local Government Board not only showed great sympathy with the object of the deputation, but, we are glad to say, displayed some serious intention of tackling this all-important problem on the lines suggested by the Conference. Mr. Burns, who is also president of the Conference, was able to assure the deputation on behalf of his colleagues in the Government that they were prepared to accept the recommendations of the deputation generally, and that he would personally enquire into the question of infant feeding and deal with it by a departmental order, or by legislation if necessary. Both the Premier and Mr. Burns rightly laid stress on improper housing and sanitation and dirt, and drink as important contributing factors to high infant mortality; yet they were not equally zealous to deal practically with these latter questions when they were approached recently by a deputation from the National Housing Reform Council. Mr. Burns referred to the responsibilities of the local authorities and to their apathy in carrying out their duties under the Public Health and Housing Acts, and Sir Henry Campbell-Bannerman drew a comparison between the care we bestow on the working man's health in the factory and our neglect of his home surroundings. It is precisely this difference between the administration of the Housing Acts and the Factory Acts that induced the Council to recommend that a statutory duty should be laid on all local authorities to appoint properly qualified medical officers of health and sanitary inspectors, who should not be removable except with the consent of the Local Government Board. It is not to be expected that a medical officer will ride roughshod over the local authorities, who are in all probability personally interested in a policy of *laissez-faire*, when he is dependent upon their good-will for his annual re-election. The deputation received, however, no encouragement to expect any effort on the part of the Government to give this necessary safeguard to medical officers of health.

